# Propolis: Properties, Application, and Its Potential

**DOI:** 10.1155/2013/807578

**Published:** 2013-08-12

**Authors:** Wojciech Król, Vassya Bankova, José Maurício Sforcin, Ewelina Szliszka, Zenon Czuba, Andrzej K. Kuropatnicki

**Affiliations:** ^1^Department of Microbiology and Immunology, Medical University of Silesia, 41-808 Zabrze, Poland; ^2^Institute of Organic Chemistry, Centre of Phytochemistry, Bulgarian Academy of Sciences, 1113 Sofia, Bulgaria; ^3^Department of Microbiology and Immunology, Biosciences Institute, UNESP, 18618-000 Botucatu, SP, Brazil; ^4^Pedagogical University of Krakow, 31-128 Krakow, Poland

Propolis is a honeybee product known for its biological and pharmacological properties for centuries. It has been extensively used in traditional medicine and also, because of its antibacterial, antiseptic, anti-inflammatory, and anesthetic activities, in complementary medicine. Propolis became subject of numerous studies developed and carried out all over the world in order to analyze its chemical composition as well as medicinal properties. 

The inspiration for this special issue was the tenth anniversary of death of Professor Stan Scheller, a precursor of propolis research in Poland. For this special issue we invited investigators and scholars to submit original research reports and review articles as well as short communications on the topic of propolis: its history, the history of the research on propolis, chemical composition of propolis, activity of propolis, and application of propolis in medicine, dentistry and veterinary medicine. More than 50 papers were submitted from which we have selected 41 which represent the rich and multifaceted knowledge. They cover a wide range of topics and are divided into review and research articles.

The research articles provide background and are the starting point for discussion of research trends in general terms. They deal with historical aspects of propolis research and focus on Professor Scheller's pioneer studies on propolis that commenced in Poland in the early 1960s. It was Scheller and his team who developed a method of introducing ethanol extracts of propolis into aqueous solutions. They showed that propolis acts as antioxidant and radioprotector, stimulates regeneration of tissue, and has immunomodulatory properties. Another paper describes Scheller's achievements in applying propolis in the treatment of burns, venous ulcerations, suppurative osteitis, and arthritis as well as postoperative wound complications. 

Some authors describe advances in the studies on chemical composition of propolis as well as botanical sources resulting in its geographically conditioned diversity. In another paper the authors present developments in the analysis and pharmacological properties of propolis which are the starting point for preparation standardization using as an example Romanian propolis. Practical applications of propolis in medicinal therapy and cosmetics are also reviewed. A separate group of papers deals with prophylactic and medicinal properties of propolis preparations in the treatment of cardiovascular diseases, cancer, oral diseases, and wound healing. 

Most of the articles in this special issue are of research character. They present the results of a variety of studies comprising different propolis extracts and their fractions as well as chemical compounds isolated from them. The articles describe experimental studies, both *in vitro* and *in vivo*, and clinical studies. 

One group of articles deals with anticancer properties of propolis. The effect of caffeic acid phenethyl ester (CAPE) on epithelial-mesenchymal transition (EMT) of human pancreatic cancer cells was investigated. The authors conclude that CAPE could inhibit the orthotopic growth and EMT of pancreatic cancer cells. The assessment of cytotoxic action of geopropolis produced by stingless bees on canine osteosarcoma cells showed that it was efficient against OSA cells in a dose- and time-dependent way. Bioactive fraction of geopropolis was also shown to decrease neutrophils migration in inflammatory process. Other authors showed that nymphaeol-A, the major component of Okinawan propolis, suppresses angiogenesis and that is why it may be a useful agent for preventing tumor-induced angiogenesis. In other studies, anticancer activity of the ethanol extract of Indian stingless bee propolis was explored by testing the cytotoxic and apoptotic effects in four different cancer cell lines at different concentrations. It was demonstrated that antioxidant potential of Indian stingless bee propolis substantiates its anticancer activity.

Another group of articles deals with anti-inflammatory and immunomodulatory activity of propolis. Some researchers showed that Brazilian green propolis and Chinese (poplar type) propolis demonstrate anti-inflammatory property. Brazilian green propolis extract proved to be effective in regulating inflammasomes which are formed in the cell cystol in response to stress signals, toxins, and microbial infections. It also showed a direct action against parasite and displayed immunomodulatory effects on murine macrophages. 

A separate group of articles deals with wound healing. Propolis proved effective in decreasing the amount of free radicals in burn wounds. Propolis burn treatment led to enhanced collagens and its components expression. Application of propolis ointment for topical treatment of nonhealing venous leg ulceration accelerated the healing process. Other studies demonstrated the ability of propolis phenolic acids and vanillin to penetrate into skin epidermis and dermis and thus to contribute to skin protection from free radicals formed under UV and premature skin aging. Also Brazilian green propolis which contains biocellulose membranes demonstrates antimicrobial activity and wound healing properties and as such is a promising biomaterial for skin wound healing. From another paper it is clear that propolis accelerates chondroitin/dermatan sulfates structure modification responsible for binding growth factors which play a crucial role in the tissue repair. 

Use of propolis in dentistry is the topic of another group of articles. The exposure of *Streptococcus mutans* and *Lactobacilli* isolated from saliva to ethanol extract of Polish propolis showed its antibacterial effect. Brazilian green propolis demonstrated a similar effect to miconazole in the treatment of *Candida*-associated denture stomatitis. A toothpaste containing propolis was found to be effective in improving oral health and treatment of gingivitis caused by dental plaque. A good effect on the health of oral cavity was also demonstrated when studying the application of toothpaste containing ethanol extract of Brazilian propolis. EEP had a positive influence on hygiene, gingival condition, and oral microflora in patients with cleft lip and palate treated with fixed orthodontic appliances. 

Propolis was shown to have antidepressant-like properties, antimicrobial activity, cytotoxic activity, and protective effect against liver damage with cholestasis. Polyphenols that are contained in propolis possess immunomodulatory, chemopreventive, and antitumor effects. They exert their chemopreventive effect by multiple molecular mechanisms on apoptosis signaling pathways in cancer cells. EEP and polyphenols isolated from propolis have been shown to sensitize cancer cells to TRAIL-induced apoptosis.

We envision that this special issue will arise more interest in propolis and more interesting investigations will be conducted.

